# Impact of Frying Olive Oil Type on the Physicochemical and Sensory Quality of Commercial Chicken Nuggets

**DOI:** 10.3390/foods14193315

**Published:** 2025-09-24

**Authors:** Tatiana Pintado, María Dolores Álvarez, Beatriz Herranz, Gonzalo Delgado-Pando

**Affiliations:** 1 Instituto de Ciencia y Tecnología de los Alimentos y Nutrición (ICTAN), CSIC, 28040 Madrid, Spain; tatianap@ictan.csic.es (T.P.); mayoyes@ictan.csic.es (M.D.Á.); herranz@ucm.es (B.H.); 2 Department of Food Technology, Faculty of Veterinary, Complutense University, 28040 Madrid, Spain

**Keywords:** rate-all-that-apply, volatiles, olive pomace oil, frying oils, extra virgin olive oil, texture sound

## Abstract

Frying is one of the most widely used cooking techniques, and olive oil is considered a suitable medium due to its high monounsaturated fatty acid content and natural antioxidants. Different olive oil categories vary in quality and price, yet their impact on fried food quality remains underexplored. This study used commercial chicken nuggets, a product commonly fried at home (180 °C), to evaluate how extra virgin, refined, and pomace olive oils influence nutritional (moisture, fat, and fatty acids), physicochemical (mechanical and acoustic properties, colour, and volatiles), and sensory attributes (Rate-All-That-Apply and hedonic tests). Overall, oil type produced minimal differences. Fat content did not vary (18.00–18.58 g/100 g), and although some fatty acid differences were significant, they were nutritionally negligible. Instrumental colour and most texture parameters were also unaffected. Volatile analysis showed terpenes as the most abundant class, with significantly higher levels in nuggets fried in extra virgin olive oil (344.8) compared with refined and pomace oils, which were similar (218.6 and 172.8, respectively). Nuggets fried in pomace olive oil were more often associated with toasted and burnt notes, supported by higher pyrazine levels (124.8 vs. 80.1 and 33.5), yet overall liking did not differ significantly (6.4 vs. 6.7 and 6.8). These results suggest that pomace olive oil, being considerably more affordable, represents a cost-effective frying alternative without compromising product quality.

## 1. Introduction

Olive oil (from *Olea europaea* L.) plays a central role in the Mediterranean diet, commonly used both as a salad dressing and as a cooking medium, particularly for frying. Although there are up to eight categories of olive oil, only a few are marketed for direct consumer use: extra virgin olive oil (EVOO), virgin olive oil, refined olive oil blended with virgin oils (commercially labelled as “olive oil”), and olive-pomace oil [1]. These categories differ in physicochemical parameters (e.g., free acidity, peroxide value, fatty acid profile, sterol composition) and in organoleptic quality. Olive oil is rich in monounsaturated fatty acids (typically ~70–75%), with lower proportions of saturated (~15–20%) and polyunsaturated (~10–12%) fatty acids which together with inherent antioxidant compounds make it relatively stable under heat, a desirable feature for frying, and it is considered to have potential health-promoting properties [2,3,4].

Frying remains one of the most widely employed culinary techniques due to the unique sensory attributes it imparts—enhanced flavour, appealing colour, and a desirable crispy texture—all of which contribute to its broad consumer acceptance [5]. Technically, frying is a rapid dehydration process where water from food is partially replaced by oil, leading to the formation of a crust that subsequently limits further oil absorption [6]. Chicken nuggets are among the most popular fried foods worldwide, consumed both at home and through commercial/frozen products, making them a familiar and suitable model for studying frying effects [7]. Although not optimal from a nutritional perspective, frying can act as a vehicle for lipid-soluble nutrients such as essential fatty acids and phenolic compounds [5]. On the other hand, the high temperatures involved in frying, combined with the presence of oxygen and moisture, trigger chemical reactions such as oxidation, hydrolysis, and polymerisation, which degrade the oil and directly affect the quality of the final product. The extent of these changes depends largely on the type and quality of the frying medium [8]. Olive oil is considered a suitable option due to its high content of monounsaturated fatty acids and protective antioxidants such as α-tocopherol, phenolic compounds, squalene, and Δ5-avenasterol [6]. Nevertheless, the potential benefits of frying must always be balanced against the increased energy density of fried foods, underscoring the importance of moderation within a healthy diet.

Beyond health and culinary considerations, the promotion of olive oil consumption holds strategic importance within the European context, as Europe is the largest producer, consumer, importer, and exporter of olive oil worldwide. Olive oil production represents a key component of the agri-food economy in several Mediterranean countries, particularly Spain, Italy, and Greece, which together account for the majority of global olive oil output. The European Union alone produces approximately 68% of the world’s olive oil [9]. However, in recent years, the consumption of EVOO has declined, partly due to sharp price increases of up to 85% in some cases. This price escalation directly affects consumers, who increasingly turn to alternative categories. For instance, in week 35 of 2025, Spanish market prices (EUR per 100 kg) for EVOO, refined olive oil, and pomace olive oil were 394.79, 322.83, and 212.98, respectively [10]. Such differences are not trivial, as price gaps influence purchasing decisions and overall consumption trends. In this context, encouraging the use of all olive oil categories, including refined and pomace oils, is essential to support the sector. Stimulating demand across the full spectrum of olive-derived oils can enhance the resilience, sustainability, and global competitiveness of the industry, while also aligning with EU policies to preserve traditional foods and foster regional agricultural development.

Based on this context, the present study uses commercial chicken nuggets as a model product commonly fried at home to evaluate how different olive oil categories (extra virgin, refined, and pomace) influence their nutritional, physicochemical, and sensory properties.

## 2. Materials and Methods

### 2.1. Materials

Extra crispy commercial nuggets (FindusEspaña, Madrid, Spain) were selected for the frying trials. According to the supplier, the ingredients of the nuggets were chicken breast 29.9%, water, seasoned breadcrumbs (wheat flour, water, spices, aromatic herbs, yeast, and salt), sunflower oil, breadcrumbs, wheat flour, fried onion (onion and sunflower oil), extruded wheat (wheat flour, dextrose, and salt), wheat starch, salt, milk proteins, and spices. Their raw composition is showed in Table 1. Three types of olive oil, all of them from the same brand, were used: extra virgin olive oil, refined olive oil, and olive pomace oil (blend of refined olive pomace oil and virgin olive oils) (La Masía, Seville, Spain). All ingredients were purchased from a local supermarket.

### 2.2. Experimental Design and Pan-Frying of the Nuggets

Three different frying batches per type of oil were used, ensuring a true triplicate. Each batch consisted of three pieces of nuggets that were pan-fried in 200 mL of oil fully covering the pieces. The samples were named as follows: NO nuggets fried in refined olive oil, NP nuggets fried in olive-pomace oil, and NV nuggets fried in EVOO. To account for variability in the frying process, nuggets from the different batches were selected for each analysis. Regardless of the oil type, the frying medium was preheated to approximately 90 °C. Once the target temperature was reached (180 °C), nuggets were fried for 5 min, being turned every minute and, during the final minute, every 30 s. This procedure ensured that the thermal centre of the product reached 70 °C.

### 2.3. Physicochemical Characterisation of Nuggets

#### 2.3.1. Moisture and Fat Composition

Moisture was determined on fried nuggets by oven-drying at 105 °C to constant weight [11] and expressed as a percentage relative to the fresh weight. The total fat level and the fatty acid profile were also calculated. The fatty acid composition was determined in freeze-dried (Cryodos Lyophilizer Equipment, Telstar, Terrasa, Spain) fried nuggets, performed (in triplicate) by gas chromatography, as reported by Pintado et al. [12]. Results were expressed as g/100 g of the product using a semi-quantitative approach with C13:0 as the internal standard. The total fat level of fried products was calculated based on the lipid conversion factors for calculating the fatty acid contents of foods [13].

#### 2.3.2. Cooking Performance and Instrumental Colour

Cooking performance was evaluated by measuring water and fat losses, as well as the size reduction experienced by the nuggets. Losses were calculated by the difference in weight before and after cooking and tempering the products to room temperature (20 ± 3 °C).

**Figure 1:**
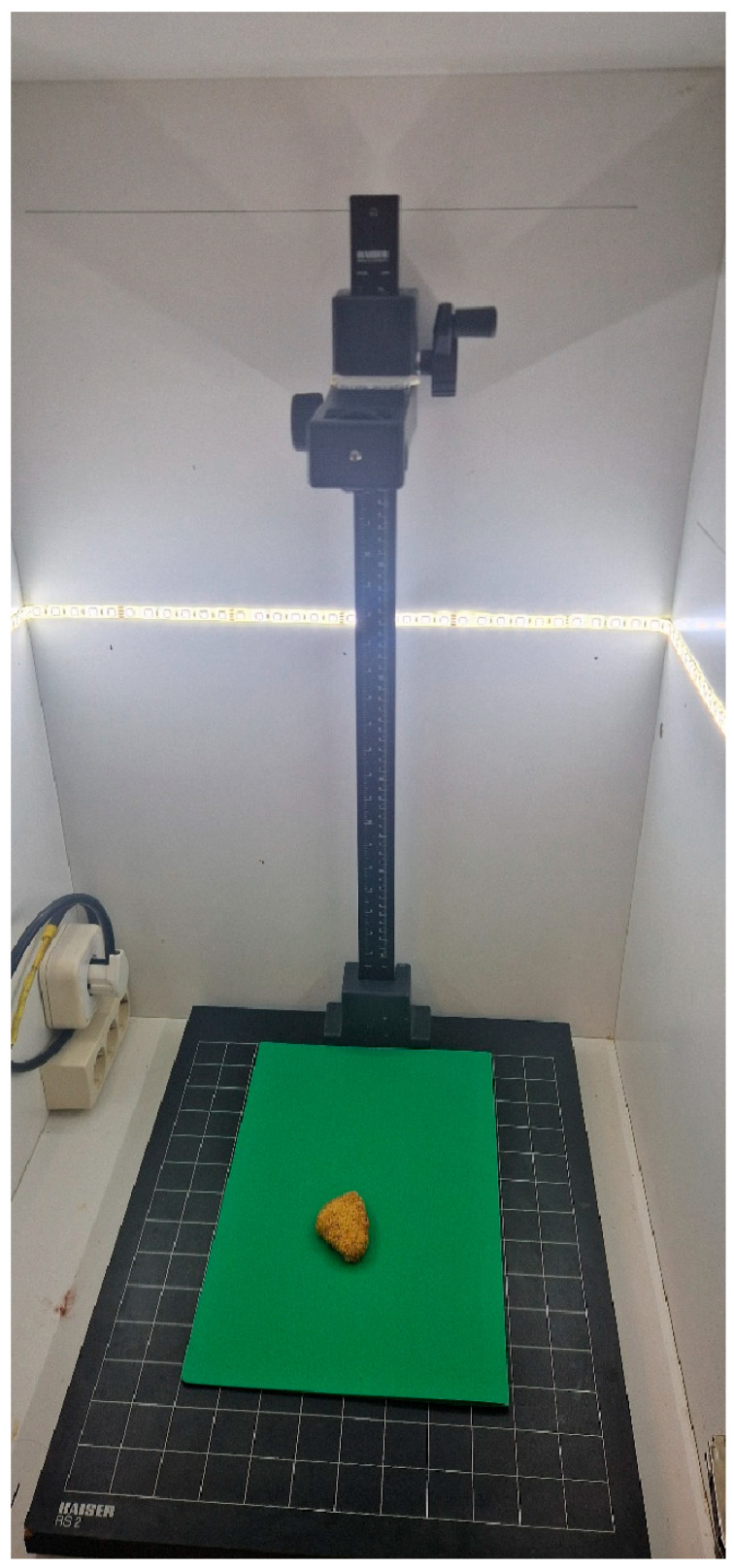
Light-controlled imaging cabinet for colour and size determination.

For size reduction and colour determination, a mirrorless camera was utilised (Nikon Z50, Tokyo, Japan) with a 16–50 mm lens (Nikkor Z DX 16–50 mm f/3.5–6.3 VR, Tokyo, Nikon, Japan). For the sampling, a light-controlled imaging cabinet was used that included LED illumination (5000 K) and a fixed stand (Figure 1). Pictures were taken of uncooked and cooked nuggets over a green background card with the camera fixed at a height of 70 cm and with the following parameters: 50 mm; ISO 160; F6.3; aperture 1/40. The images were stored and verified before being processed. From each image, perimeter and area were calculated using Python 3.13 (The Python Software Foundation, Beaverton, OR, USA) and the following packages: OpenCV [14], NumPy [15], pandas [16], and scikit-image [17]. First, the correct pixel-to-mm scale was set, colour thresholding was applied to remove the green background, and then the object information was extracted: perimeter, area, and mean R, G, and B values from the RGB colour space.

#### 2.3.3. Instrumental Texture and Sound Emission Analysis

A TA.HDPlus Texture Analyzer (Stable Micro Systems (SMS), Ltd., Godalming, UK), equipped with a 5 kg load cell and with Texture Exponent Software (6,2,6,0 version), was used for obtaining the force/distance measurements. A cutting test was performed using a craft knife adapter (A/CKB; SMS Ltd., Godalming, Surrey, UK), which accommodated a standard 50 mm wide craft blade. Each nugget piece was cut three times, through its top, centre, and bottom, up to a depth of 8 mm at a compression speed of 1 mm/s, with a data acquisition rate of 500 points/s for the force signal. The average of the three measurements was taken as representative of the texture of each nugget. Mechanical parameters derived from each cutting test were as follows: maximum force (Max F, N), area under the force–distance curve (Work FD, mJ), number of force peaks (calculated for a drop in force higher than 0.049 N, Peaks F, dimensionless), and average drop off (calculated as the average drop in force between consecutive peaks and troughs, Drop off F, N).

The sound emitted during the cutting test of nugget samples was simultaneously recorded by an acoustic envelope detector (AED), as described in detail elsewhere [18]. The gain of the AED was set at 0 (0 dB). Ambient acoustic and mechanical noise was filtered with the use of an envelope corner frequency filter of 3.125 kHz. The data acquisition rate was also 500 points/s for the acoustic signal. From the sound curves, the following acoustic parameters were determined in accordance with previous studies; [18]: the maximum of AED sound peaks (maximum value of sound pressure level (SPL), Max peak SPLmax, dB), the number of AED sound peaks (calculated for a drop in SPL larger than 2 dB, Peaks AED, dimensionless), the mean of AED sound peaks (the sum of the peaks values divided by number of the peaks, Mean AED peaks, dB), the area under the distance–acoustic signal AED curve, area AED, dB mm), and average drop off (calculated as the average drop in sound between consecutive peaks, Drop off AED, dB). Three individual nuggets per batch and each frying oil type were utilised.

#### 2.3.4. Volatile Determination

Volatile compounds of the fried nuggets were extracted using headspace solid-phase microextraction (HS-SPME) and identified using a gas chromatograph (Agilent, model 7890, Santa Clara, CA, USA) equipped with a 5975 Mass Triple Axis Detector. We followed the protocol as in [19]. Briefly, fried nuggets samples were homogenised in a blender and 5 g was used for the volatile analysis. The samples were placed in a 20 mL glass vial sealed with an aluminium cap and septum, then incubated at 40 °C for 30 min. SPME was carried out using a Divinylbenzene/Carboxen/polydimethylsiloxane fibre (50/30 µm, 2 cm; Supelco) common in volatile analysis of similar foodstuffs such as nuggets [19] and fried dough [20]. It was preconditioned at 270 °C for 1 h as per the manufacturer’s instructions. The fibre was exposed to the headspace for 30 min at 40 °C, then desorbed in the GC injection port for 30 min. For the volatile separation, a DB-WAX UI polyethylene glycol capillary column (60 m, 320 × 0.25 μm) was used. The operating conditions were as follows: the oven temperature was set initially at 40 °C (4 min hold), increased to 110 °C at 4 °C/min, to 180 °C at 6 °C/min, and to 240 °C at 8 °C/min (15 min hold). Helium was used as a carrier gas at 1.3 mL/min; injector and detector temperatures were held at 250 °C and 240 °C, respectively. The mass spectra were obtained using a mass selective detector collecting data at a rate of 4 scan s^−1^ over the m/z range of 30–360. An n-alkanes mixture (Supelco Alkane standard solution C8–C20) was analysed under the same conditions in order to obtain the linear retention indexes (LRIs). Each volatile compound was identified by comparing mass spectra and RI value with mass spectra libraries (Wiley Registry 11th edition/NIST 2017 library) and the literature.

### 2.4. Sensorial Analysis

The sensory test was carried at CSIC-ICTAN’s facilities in individual sensory booths with standard lighting and temperature. A total of 56 adult panellists (39 female) participated in the analysis. They were voluntary staff members familiar with this type of testing and consumers of fried products. They filled up the sociodemographic and sensory questionnaires using individual tablets and the data were collected using Sensesbit software v4.5.1 (Lugo, Spain).

Participants evaluated each sample during a two-step procedure: First, a Rate-All-That-Apply (RATA) test was conducted followed by the assessment of the overall liking (9-point hedonic scale). For RATA, panellists were instructed to select all attributes that were applicable for describing the sample and to rate the intensity of the applicable terms using a 3-point scale: ’low’, ’medium’, and ’high’. If any attribute was not applicable to the sample then a 0 was chosen instead. The attributes were based upon prior research and pilot work with a selected team of panellists to help select descriptors that better discriminated the samples. A total of 17 sensory attributes were used: 4 regarding appearance (toasted appearance, visual crunchy, external homogeneity, and internal homogeneity), 3 regarding odour (spices odour, off odour, and fried odour), 4 regarding texture (crunchy exterior, juiciness, chewability, and oily texture), and 6 regarding flavour (chicken flavour, oily flavour, spices flavour, burnt flavour, off flavour, and acid aftertaste).

In each test, panellists were presented with three samples, coded with three-digit numbers in a randomised fashion to cover all possible combinations. Water was provided to panellists to neutralise flavours between samples.

### 2.5. Statistical Analysis

All data were processed using R 4.4.3 [21] in R Studio [22]. The effects of frying oil on the physicochemical characteristics of the nuggets was analysed using a one-way ANOVA and Tukey’s HSD post hoc comparison for those that were significant (*p* < 0.05). As suggested by Meyners et al. [23], RATA and hedonic testing data was first analysed by means of a mixed ANOVA where the type of frying oil acted as a fixed effect and panellist as a random effect; the Holm method was used for post hoc pairwise comparison of those significant attributes. Principal Component Analysis (PCA) was then applied to the RATA mean scores to obtain a graphical distribution of sample and attributes. The following R packages were utilised: tidyverse [24], rstatix [25], factomineR [26], and factoextra [27].

## 3. Results and Discussion

### 3.1. Moisture and Fat Composition of Fried Nuggets

The moisture and fat content of the fried nuggets were similar, approximately 40% and 18%, respectively (Table 2). According to the nutritional information of the raw nuggets (Table 1), which indicates a total fat content of 11 g per 100 g of product, it can be observed that the fat level increases (proximately to 63%) during the frying process to a similar extent regardless of the type of olive oil used. This behaviour is consistent with the frying mechanism, which causes dehydration of the product accompanied by the absorption of oil from the frying medium [5]. However, these commercial nuggets absorbed more fat than that reported for chicken nuggets subjected to frying in high-linoleic and high-oleic sunflower oils, which exhibited only a 17% increase in fat content compared to the raw product [28].

Regarding the fatty acid profile, differences are indeed observed depending on the type of olive oil used (Table 3). Considering that the composition of EVOO, olive oil, and pomace olive oil is similar in terms of fatty acid profile [4], and that the raw product is the same, the differences in the lipid profile of the fried products can be attributed to the different behaviour of the oils during the frying process [2].

In terms of the saturated fatty acids (SFA), palmitic and stearic acids were predominant across all frying treatments. The use of extra virgin olive oil (EVOO) and olive oil for frying the nuggets resulted in higher (*p* < 0.05) SFA levels compared to frying with olive-pomace oil. As anticipated, oleic acid was the major fatty acid in all samples, reflecting its predominance in the olive oils used for frying [4] and in the chicken meat incorporated in the reformulated product [29]. Oleic acid was the most abundant fatty acid in all products (Table 3), as expected, given its predominance in the olive oils used for frying [4] and in the chicken meat [29] used as ingredient in the reformulated product. Notably, both the oleic acid content and the total monounsaturated fatty acids (MUFA) were significantly higher when EVOO was employed. Conversely, frying with olive-pomace oil yielded nuggets with higher polyunsaturated fatty acids (PUFA) levels (Table 3), along with an improved profile of both n-3 and n-6 fatty acids. The observed shift in fatty acid composition, while statistically significant, is unlikely to hold nutritional relevance given the very small magnitude of the differences.

### 3.2. Cooking Performance and Instrumental Colour Determination

Cooking performance was evaluated based on dimensional changes, calculated from the shrinkage of the nuggets, as well as the weight loss occurring as a consequence of the frying process. The type of oil used for frying did not significantly affect the cooking performance of the product (Table 4). The surface area of the nuggets decreased by 4.8–5.2% compared to the fresh products. In terms of weight loss, nuggets fried in olive-pomace oil showed a 12.3% reduction, whereas those fried in extra virgin olive oil (EVOO) exhibited a 13.3% reduction. Several authors have found a direct linear relationship between shrinkage and moisture loss of different foods, nuggets included, during frying processes [30]. On the other hand, given that the relationship between moisture content and oil absorption is inversely proportional, a significant loss of moisture generally results in a corresponding increase in oil uptake by the food product [7]. The observed losses therefore highlight the ability of the nuggets to absorb oil during frying. From a nutritional perspective, this could be advantageous, as a substantial proportion of the bioactive compounds naturally present in the frying olive oils [4] are retained in the final product consumed.

Frying inherently induces characteristic colour changes in foods as a result of water loss, oil uptake, and thermally driven reactions such as Maillard browning and pigment degradation. This process leads to progressive darkening and browning, which serves both as a marker of product quality and as a key determinant of consumer acceptance [31]. In our study, no significant differences were observed in overall colour development between nugget samples. All fried nuggets showed a decrease in R, G, and B values compared with their raw counterparts, but these reductions were consistent across samples (Table 5). This trend aligns with previous reports in which frying decreased nugget colour intensity [32,33]. To further investigate whether frying oil could influence localised colour patterns, we classified nugget surfaces into three luminosity ranges: light (lightness < 15), medium (15 ≤ lightness ≤ 43), and dark (lightness > 43). Comparison of raw and fried nuggets revealed that frying significantly increased (*p* < 0.01) medium and dark areas by an average of 1317.32 and 53.42 mm^2^, respectively, while significantly decreasing (*p* < 0.01) the light area by 1463.97 mm^2^. However, no significant differences were detected among the different frying olive oil types.

### 3.3. Mechanical and Acoustic Properties

**Figure 2:**
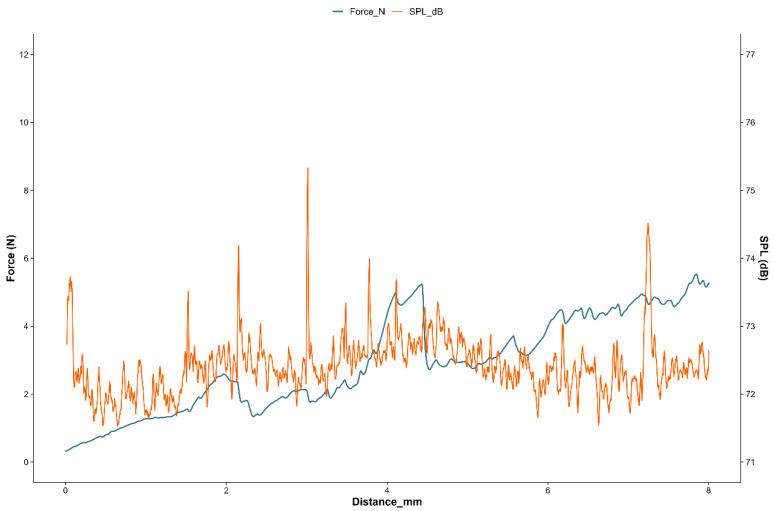
Typical mechanical (force—blue line) and acoustic (SPL—orange line) parameters for a fried nugget.

Mechanical and acoustic attributes are key indicators of crispness in coated fried foods and can be objectively assessed by simultaneous force–distance and sound pressure measurements [32]. Typical profile of the fried nuggets (Figure 2) showed jagged curves with numerous force and sound peaks, many of which coincided.

Table 6 presents the effect of frying oil type on the instrumental texture parameters obtained from a cutting test. Among the mechanical parameters, oil type had a significant effect only on the work values (Work FD) derived from the force–distance curves. Nuggets fried in virgin olive oil (NV) exhibited the highest Work FD values, while those fried in pomace olive oil (NP) showed the lowest. The number of force peaks (Peaks F) recorded in this study was higher than those reported by other authors for commercial chicken nuggets [34], indicating that our commercial nuggets possess an adequate external breadcrumb coating, which likely contributes to a greater number of fracture events during cutting.

Regarding the acoustic parameters, a significant effect of oil type was observed only for SPL_max_, with the highest values recorded in nuggets fried in virgin olive oil (NV) and the lowest in those fried in refined oil (NO). These SPL_max_ values were also higher than those reported by Albert et al. [35], further supporting the presence of an adequate coating layer that produces a louder sound when the nuggets are broken by cutting. Additionally, Oğraş and Kaplan [36] evaluated the instrumental texture properties of turkey nuggets fried in sunflower and hazelnut oil and found a negative correlation between hardness and moisture loss during frying under atmospheric conditions, but only in the case of hazelnut oil.

### 3.4. Volatile Compounds

Understanding how different frying methods influence the formation of volatile compounds is crucial for optimising the sensory profile of fried foods and enhancing consumer acceptance [37]. In our study, we assessed the effect of different olive-type frying oils on the volatile composition of commercial nuggets.

A total of 78 volatile compounds were identified (Table 7). The number of volatiles detected significantly varied depending on the frying oil used (F = 8.857, *p* = 0.016). Nuggets fried in NO oil had the lowest number of volatiles (57), which was significantly different from the 69 compounds found in NV samples. Nuggets fried in NP oil showed an intermediate value (60 compounds), not statistically different from either NO or NV, as indicated by Tukey’s HSD post hoc test. When analysing the number of compounds per chemical class, no significant differences were found among the three sample types (χ^2^ = 7.77, *p* = 0.999). Overall, the most abundant group was sulphur-containing compounds (12–13 identified), followed by terpenes (9–11), aldehydes (7–8), aliphatic (6–8), pyrazines (2–8), ketones (4–7), furans (3–5), esters (2–4), and acids (2–3). Additionally, single representatives of alcohols, nitrosamines, pyrimidines, and benzenes were also identified.

Analysis of total peak areas revealed significant differences among samples, both at the compound-type level and for several individual volatiles (Table 7). Among the chemical classes, terpenes were the most abundant and showed significant variation, with the highest abundance in the NV sample, significantly higher than in NO and NP. Terpenes are natural hydrocarbons composed of connected isoprene units and mainly found in fruits, vegetables, and herbs as secondary metabolites, but are not major constituents of olive oils [38,39]. In our case, the commercial nugget recipe included a great quantity of spices likely accounting for their presence. The significant difference between the samples, although seemingly counterintuitive, may be explained by the antioxidant capacity from EVOO even during frying [40] that could have reduced the thermal degradation of spice-derived terpenes and by-products that could interfere with terpene release to the HS. These factors suggest that EVOO provides a frying environment that better preserves terpenes, highly sensitive to heat and oxidation. At the individual level, 3-Carene and D-Limonene followed the same pattern, being higher in NV than in NO and NP. These terpenes are typically associated with black pepper and white pepper, respectively [41]. On the other hand, the rest of the individual terpenes were mostly significantly higher in NV but only when compared to NP. The profile of terpenes detected aligns with previous findings in nuggets prepared from commercial spice mixes [19].

The total aldehyde abundance did not significantly differ between samples. Aldehydes are secondary products from lipid oxidation often associated with off-flavours and off-odours in meat and are considered reliable markers of lipid oxidation [42]. In our study, the most abundant aldehyde across all samples was hexanal, followed by 2-methylbutanal and 3-methylbutanal, both formed by Strecker degradation of isoleucine and leucine, respectively. These Strecker aldehydes are important aroma contributors in various foods, typically imparting malty and yeasty notes [43] and even contributing to desirable aroma notes in cooked products, such as the ‘toasted’ flavour [19]. Hexanal, a major volatile in lipids rich in linoleic acid (such as olive oil), is widely recognised as a key indicator of lipid oxidation in meat products, particularly those high in n-6 fatty acids [44]. Pentanal, another lipid oxidation marker, was not detected in NP, whereas propanal, often linked to n-3 fatty acid oxidation, was present in all three samples (Table 7). Among the aldehydes, only 2,4-decadienal presented a significantly higher abundance in NV than in NP and was not detected in NO. This compound is prone to further oxidation and can decompose further to hexanal, pentane, acetic acid, and hexanoic acid [42], all of which were also detected in the samples. Importantly, 2,4-decadienal is also considered one of the key contributors to the characteristic deep-fat fried flavour. On the other hand, aldehydes, particularly the unsaturated ones such as propanal, pentanal, hexanal, and nonanal, are often associated with rancid, painty, or beany off-flavours in lipid-rich foods [45].

Sulphur-containing volatiles play a pivotal role in meat aroma, arising from both aliphatic and heterocyclic structures, and have been spotlighted for their ability to lend meat-like characteristics to flavour systems [46]. Additionally, the allium vegetable family, especially onion and garlic, contributes significantly to sulphur compounds, and our nuggets include both, which is likely the source of the elevated sulphur profile observed [43]. In our analysis of the samples, we identified 13 distinct sulphur compounds, with no significant differences between frying oils at either the group or individual compound level. Remarkably, methyl-thiirane (also known as propylene sulphide) was the most abundant volatile overall, not only within the sulphur class but across all volatiles detected (Table 7). This compound, also known as propylene sulphide, has been found in cooked beef, roasted chicken, and garlic [43,47]. We also detected key allium-derived sulphur volatiles such as diallyl disulphide, methanethiol, and methyl 2-propenyl disulphide. Diallyl disulphide, produced by the thermal degradation of allicin, is a major contributor to the pungent garlic odour and widely acknowledged as a characteristic species-level flavour compound [48]. It has also been reported in chicken nuggets [49]. Methanethiol, known for its intense cabbage-like and roasted sulphur notes in cooked foods [43], has likewise been detected in chicken nuggets [19] and is present in our samples.

Total pyrazine abundance showed significant differences between the samples, with NP exhibiting the highest levels, followed by NV and then NO. Notably, only two pyrazines, methyl pyrazine and 2,6-dimethyl pyrazine, were detected in NO, whereas 7 and 8 different pyrazines were identified in NV and NP, respectively. These marked differences point to a potential interaction between the frying oil and the nugget matrix that may have influenced Maillard reaction pathways. Pyrazines are well-known Maillard reaction products, commonly formed in meat and roasted foods (such as bread) under heat and are not typically associated with heated olive oils [38,43,50]. Hence, meat component and breadcrumbs from the commercial nugget recipe are the main contributors to pyrazine content.

Their formation depends on several factors, including the presence of amino acids and reducing sugars, pH, temperature, and lipid interactions. The higher pyrazine levels in NP and NV suggest that frying in pomace or extra virgin olive oil may have either promoted their formation or enhanced their extraction into the headspace during SPME-GC-MS analysis. Among all pyrazines, methyl pyrazine was the most abundant in all samples. However, its levels were also significantly different across oils, with NP > NV > NO; this trend was observed for each individual pyrazine. Pyrazines are key aroma compounds in many cooked foods, contributing to roasted, nutty, and earthy notes. While methylated pyrazines are common in meat volatiles, their relatively high odour thresholds (>1 mg/kg) limit their sensory impact. In contrast, ethylated pyrazines, such as ethyl pyrazine, 2-ethyl-6-methylpyrazine, and 2-ethyl-3-methylpyrazine, all detected in NP and NV, exhibit significantly lower thresholds and are therefore considered potent contributors to burnt, nutty, and butterscotch-like aromas [43]. 4,6-dimethylpyrimidine, another nitrogen compound, was detected exclusively in samples fried with extra virgin olive oil (EVOO), but not with refined or pomace olive oils. This may be related to the presence of phenolic compounds or other minor constituents in EVOO that modulate Maillard reaction pathways or protect intermediates from further degradation. 4,6-dimethylpyrimidine has been reported in heated extracts of water-soluble beef flavour precursors and in roasted Dongding oolong tea [51,52].

Five different furans were detected in both NP and NV samples, whereas only three were found in NO. Total furan abundance was significantly lower in NO compared to NP and NV (Table 7), suggesting possible differences in the formation pathways of these compounds. Furans typically originate from the degradation of carbohydrates (via the Maillard reaction), thermal oxidation of lipids, thiamine degradation, or the breakdown of 5′-ribonucleotides [43]. Among them, 2-pentylfuran and 2-methylfuran are known for their beany and grassy odours, although the odour threshold of 2-methylfuran is reported to be 1000-fold higher than that of its 2-pentyl homologue. Notably, 2-pentylfuran is considered a key contributor to the reversion flavour in soybean oil and has been previously identified in chicken nuggets [19,53]. Furans such as furfurals and furanones are associated with caramel-like, sweet, and fruity notes [43]. In our study, furfural was the most abundant furan across all samples, but its concentration was significantly lower in NO. This compound, often found in the crumb of white bread, contributes smoky aromatic notes to foods.

No significant differences were observed in the total abundance of aliphatic hydrocarbons between the three samples, likely due to the high variability found in nuggets fried with extra virgin olive oil (Table 7). Although aliphatic hydrocarbons represent one of the largest classes of volatiles detected in meat, they are generally considered to contribute little to aroma or flavour, owing largely to their high odour threshold values [19,43]. Most hydrocarbons are formed through the thermal degradation of lipids via homolytic cleavage or autoxidation of long-chain fatty acids. Alcohols, similarly, are primarily derived from the autoxidation of polyunsaturated fatty acids. Among these, 1-octen-3-ol is a key compound frequently reported in meat volatiles. It was also detected in our samples and is known for its characteristic mushroom-like aroma with metallic undertones. However, no significant differences were found in its abundance across the different frying oil treatments. Sabikun et al. [49] previously identified 1-octen-3-ol in chicken nuggets, supporting its relevance in meat products. Interestingly, Giuffrè et al. [38] reported the formation of 1-octen-3-ol during heating of EVOO, but not in olive pomace oil, suggesting that the oil matrix itself could influence its presence or stability during frying.

Ketone abundance differed between the samples. NV had a significantly higher abundance compared with NO, and NP’s was also significantly higher than that of NO. Ketones originate from lipid degradation, with unsaturated ketones being typical of roasted chicken. Additionally, dicarbonyls such as 2,3-pentanedione are regularly found in meat products and serve as important intermediates in the formation of other volatile compounds [43]. In our samples, the abundance of this compound followed the same pattern as the total ketone abundance. Acetone was found only in NV samples; Yan et al. [54] also reported acetone in EVOO, as well as in pomace and refined olive oils. However, its absence in NO and NP in our study suggests that specific frying conditions or matrix interactions in EVOO may promote its formation or retention more effectively than in the other oils. 1-(2-furanyl)-ethanone, also known as 2-acetylfuran, is a furan-derived ketone widely used in flavourings for its sweet, balsamic, cereal, and nutty notes [43,55]. In our samples, its abundance was significantly higher in NP, followed by NV and lastly NO (Table 7), suggesting that mid-range processing oils like pomace may favour the formation or release of certain ketone-derived aroma compounds more than either refined or extra virgin oils.

Esters and acids were also found in our samples with differing abundance among them. NP and NV had a higher ester and acid abundance than NO. 2-Propynyl propionate, a propanoic acid propyl ester, was only found in NP and NV. This compound has previously been reported in commercial chorizo and raclette cheeses [56,57]. This suggests that the use of pomace and extra virgin olive oils may facilitate the retention or formation of esters associated with complex, fermented, or dairy-like notes, possibly due to their compositional or thermal behaviour during frying. Methyl acetate abundance was higher in NP compared to NO or NV. This ester, associated with sweet and fruity notes, may be present in higher concentrations in pomace oil or promoted by its specific frying conditions. Acetic acid showed a similar trend, aligning with the findings of Yan et al. [54]. Hexanoic acid, on the other hand, was found at comparable levels across all three samples and could serve as a marker of oil oxidation, as it imparts pungent and rancid odours [43,58].

In summary, the type of frying oil modulated both the formation and stability of key aroma-active volatiles in the nuggets, likely through its influence on lipid oxidation, Maillard reactions, and the preservation or degradation of spice-derived compounds. Compound classes such as terpenes, pyrazines, ketones, and esters were particularly affected. EVOO favoured the retention of delicate volatiles, while pomace oil appeared to promote the formation of others, highlighting the oil’s role in shaping the sensory-relevant volatile profile.

### 3.5. Sensory Analysis

The sensory panel consisted of 56 individuals (39 females), distributed across the following age groups: 14 participants aged 18–24, 19 aged 25–34, 9 aged 35–44, and 14 aged over 44 years. All participants reported being consumers of fried food: 14 consumed it one or more times per week, 21 several times per month, and 21 no more than once per month. Regarding the type of oil typically used for frying, 20 participants reported using refined olive oil for pan-frying, 2 used pomace olive oil, and 22 used EVOO, while the remaining 12 reported using sunflower oil or other seed oils. These preferences align with national consumption trends in Spain for olive oils, where per capita consumption in 2024 was estimated at 2.20 litres for refined olive oil, 0.29 litres for pomace olive oil, and 2.78 litres for EVOO [59], although these figures reflect overall consumption and not exclusively frying use.

RATA is a suitable approach for detecting such subtle differences in complex products, as it allows panellists to freely check all relevant descriptors, providing richer sensory profiles than traditional methods [60]. Of the 17 attributes included in the RATA questionnaire, only 3 showed significant differences between the samples (Table 8): toasted appearance, fried odour, and burnt flavour. In terms of appearance, the panellists observed that the nuggets fried in pomace olive oil had a stronger toasted colour when compared with the ones fried in refined olive oil. This difference was not confirmed by instrumental colour measurements, likely due to the greater sensitivity of human vision to localised browning and surface heterogeneity, which are averaged out in instrumental analyses. Nonetheless, the fried odour of the NP sample was rated as significantly stronger than that of NV. This was consistent with the significantly stronger burnt flavour perceived in NP, albeit at low intensity. In agreement, volatile analysis (Table 7) showed higher levels of total pyrazines in NP than NV, supporting their role as contributors to burnt notes. Interestingly, although pyrazine levels were significantly lower in NO, this difference was not detected by the panellists. This highlights the complexity of flavour perception in composite matrices such as nuggets, where interactions between volatiles and the food matrix, as well as cross-modal integration of odour and taste cues, may mask or attenuate specific compound contributions [61,62]. Although significant differences in terpene content were also found (Table 7), panellists did not report any corresponding differences in spice odour or flavour, and these attributes were rated at low intensity (Table 8). It should also be noted that the panellists in this study were consumers rather than trained assessors, which likely limited their ability to detect subtle differences in specific aroma-active compounds. No significant differences were found in the overall liking of the nugget samples with mean values close to “like moderately” for the three samples (Table 8). This shows that, at least in terms of liking, the oil type did not significantly affect the acceptance of the nuggets.

**Figure 3:**
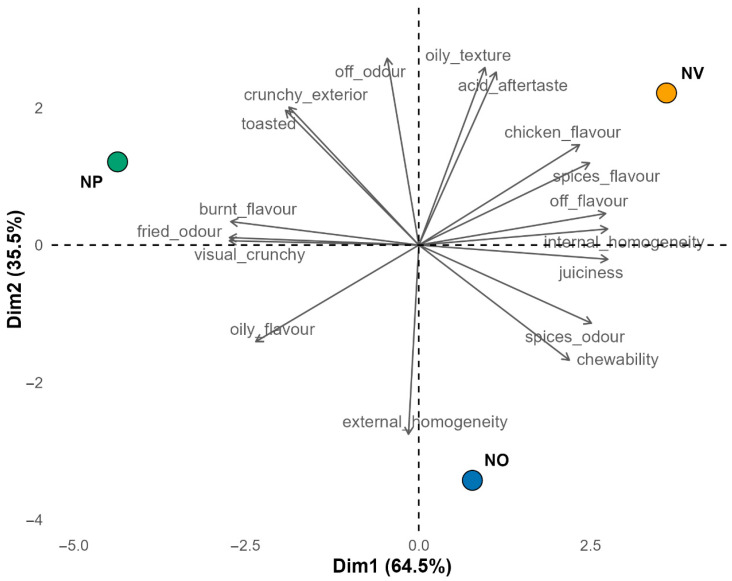
PCA biplot of RATA attributes for nuggets.

PCA was applied to the RATA dataset to reduce dimensionality and visualise relationships among samples and attributes. Arithmetic mean values were used instead of Dravnieks-type scores to emphasise intensity rather than frequency of citation, as both approaches were shown to yield very similar product maps [23]. The PCA biplot (Figure 3) shows the three nugget samples distributed across distinct quadrants, reflecting different attribute correlations. Dimension 1 explained 64.5% of the variance (eigenvalue = 10.96) and was mainly driven by visual crunchy, fried odour, juiciness, internal homogeneity, burnt flavour, and off flavour. Along this axis, NP separated clearly from the other two samples, being positively associated with fried odour, burnt flavour, and visual crunchy, but negatively with juiciness and internal homogeneity. In contrast, NV appeared on the opposite side of the axis, showing inverse correlations with those attributes and also stronger correlation with spice flavour, consistent with its higher terpene content (Table 7). The NO sample was mainly distinguished by Dimension 2 and was positively associated with external homogeneity and negatively with off odour, oily texture, and acid aftertaste. NV again showed the opposite trend, being positioned on the positive side of this dimension. These multivariate results are consistent with the univariate RATA analysis, where toasted appearance, fried odour, and burnt flavour were the main discriminating attributes, confirming that oil type slightly modulated both visual and flavour-related perceptions of the nuggets. Although the hedonic assessment revealed no significant differences, clear sensory patterns emerged: nuggets fried in pomace oil were perceived as oilier and more toasted, those fried in refined olive oil as juicier and more internally homogeneous, and those fried in extra virgin olive oil as having higher external homogeneity, spice flavour, and lower acid aftertaste.

## 4. Conclusions

This study demonstrates that the type of olive oil used for frying commercial chicken nuggets exerts only a minimal influence on their nutritional, physicochemical, and sensory characteristics. No significant differences were observed in fat content, and although some variations in fatty acid composition reached statistical significance, their magnitude was nutritionally negligible. Instrumental colour measurements and most texture parameters likewise showed no differences among nuggets fried in extra virgin, refined, or pomace olive oils. From a sensory perspective, nuggets fried in pomace olive oil were more frequently associated with toasted appearance, fried odour, and burnt flavour attributes. However, these differences were of low intensity and did not translate into significant effects on overall liking, which remained similar across all oil types. Comprehensive volatile analysis revealed differences in certain compound classes, such as terpenes and pyrazines, yet not all of these chemical variations were fully perceived by the consumer panel, underscoring the complexity of flavour perception in composite matrices such as nuggets. Taken together, these results indicate that despite minor compositional or sensory nuances, nuggets fried in extra virgin, refined, and pomace olive oils are largely comparable in terms of nutritional value and organoleptic quality. Considering that pomace olive oil is considerably more affordable than other olive oil categories, its use in frying represents a cost-effective option for consumers without compromising product quality. Considering that pomace olive oil is considerably more affordable than other olive oil categories, its use in frying represents a cost-effective option for consumers, with only minor compromises in the quality of the final product. However, further research is warranted to assess the presence and behaviour of minor compounds formed during frying, including both potentially beneficial components (e.g., phenolics) and harmful ones (e.g., polycyclic aromatic hydrocarbons), to fully evaluate its safety and functional value.

## Figures and Tables

**Table 1:** Nutritional information per 100 g of raw nuggets.

Nutritional Profile	
Total energy (Kcal)	241
Total fat (g)	11
Saturated fat (g)	1.3
Total carbohydrate (g)	26
Total sugars (g)	4.4
Dietary fibre (g)	1.4
Proteins (g)	8.8
Salt (g)	1.2

**Table 2:** Composition (g/100 g) of fried nuggets: mean (SD).

	NO	NP	NV
Moisture	39.60 (1.21) ^a^	39.85 (2.23) ^a^	39.84 (1.02) ^a^
Fat	18.00 (0.33) ^a^	18.17 (0.44) ^a^	18.58 (0.37) ^a^

For each parameter different superscript denotes a significant difference (*p* < 0.05).

**Table 3:** Fatty acid composition (g/100 g product) of fried nuggets: mean (SD).

	NO	NP	NV
Palmitic acid (C16:00)	2.49 (0.04) ^b^	2.27 (0.04) ^a^	2.42 (0.05) ^b^
Stearic acid (C18:00)	0.56 (0.01) ^b^	0.54 (0.01) ^a^	0.58 (0.01) ^b^
ΣSFA	3.25 (0.05) ^b^	3.04 (0.06) ^a^	3.20 (0.07) ^b^
Palmitoleic acid (C16:1n-7)	0.27 (0.00) ^b^	0.23 (0.00) ^a^	0.26 (0.01) ^b^
cis-Vaccenic acid (C18:1n7c)	0.41 (0.01) ^b^	0.38 (0.01) ^a^	0.43 (0.01) ^c^
Oleic acid (C18:1n9c)	10.53 (0.22) ^a^	10.80 (0.30) ^a^	11.18 (0.19) ^b^
ΣMUFA	11.26 (0.23) ^a^	11.47 (0.31) ^a^	11.91 (0.20) ^b^
Linoleic acid (C18:2n6c)	2.42 (0.04) ^b^	2.58 (0.05) ^b^	2.37 (0.07) ^a^
Linolenic acid (C18:3n3)	0.16 (0.00) ^b^	0.17 (0.00) ^b^	0.16 (0.01) ^a^
ΣPUFA	2.60 (0.04) ^b^	2.76 (0.05) ^b^	2.54 (0.07) ^a^
Σn3	0.16 (0.00) ^a^	0.17 (0.00) ^b^	0.16 (0.01) ^a^
Σn6	2.44 (0.04) ^a^	2.59 (0.05) ^b^	2.38 (0.07) ^a^

For each fatty acid or summative different superscript denotes a significant difference (*p* < 0.05).

**Table 4:** Dimensional (area and perimeter) and weight loss (%) of nuggets during cooking: mean (SD).

Sample	Area	Perimeter	Weight
NO	5.18 (0.84) ^a^	4.47 (0.68) ^a^	12.94 (0.82) ^a^
NP	4.76 (0.82) ^a^	4.81 (0.66) ^a^	12.26 (0.78) ^a^
NV	5.20 (0.90) ^a^	3.27 (1.02) ^a^	13.33 (1.27) ^a^

For each parameter different superscript denotes a significant difference (*p* < 0.05).

**Table 5:** Differences (raw–fried) in nuggets’ colour (R, G, and B) after being fried in different oils: mean (SD).

Sample	R	G	B
NO	43.18 (2.35)	64.82 (2.61)	48.21 (3.57)
NP	41.27 (5.39)	62.49 (6.18)	48.99 (3.62)
NV	40.07 (9.83)	61.48 (5.57)	43.93 (1.00)
Stats	F = 0.947, *p* = 0.439	F = 0.626, *p* = 0.566	F = 3.041, *p* = 0.122

**Table 6:** Effect of olive oil type on instrumental texture and acoustic envelope detector (AED) descriptors from a cutting test: mean (SD).

	Instrumental Mechanical Parameters				Instrumental AED Parameters				
Sample	Max F (N)	Work FD (mJ)	Peaks F * (-)	Drop off F (N)	Max Peak SPL_max_ (dB)	Peaks AED ** (-)	Mean AED Peaks (dB)	Area AED (dB mm)	Drop off AED (dB)
NO	6.86 (0.52) ^a^	31.70 (1.84) ^a,b^	19.00(1.30) ^a^	0.37(0.09) ^a^	76.75(0.44) ^a^	6.00(1.72) ^a^	75.35(0.42) ^a^	581.11(0.25) ^a^	3.53(0.41) ^a^
NP	6.78 (0.48) ^a^	27.30 (2.22) ^a^	20.00 (1.56) ^a^	0.37 (0.06) ^a^	77.21 (0.28) ^a,b^	5.00 (1.46) ^a^	74.96 (0.10) ^a^	566.74 (21.23) ^a^	3.34 (0.18) ^a^
NV	7.74 (0.53) ^a^	34.29 (1.38) ^b^	20.00 (0.68) ^a^	0.38 (0.01) ^a^	77.98 (0.16) ^b^	8.00 (0.36) ^a^	75.03 (0.24) ^a^	570.35 (5.53) ^a^	3.44 (0.05) ^a^

* Calculated for drop in force higher than 0.049 N; ** Calculated for drop in sound pressure level larger than 2 dB. Different letters within each column indicate that means are statistically different (*p* < 0.05).

**Table 7:** Volatile compounds (arbitrary area × 10^5^) found in the three nuggets after frying in different oils including olive oil (NO), pomace olive oil (NP), and virgin olive oil (NV): mean area (SD).

Compound	LRI ^1^	ID ^2^	NO	NP	NV	F-Value (*p*-Value) ^3^
Alcohol						
1-Octen-3-ol	1444	A	1.57 (1.38)	2.50 (0.63)	2.84 (0.61)	
Alyphatic						
Total alyphatic			59.83 (52.48)	40.51 (45.74)	224.54 (270.82)	
Pentane		B	10.61 (9.38)	8.39 (7.63)	11.70 (11.72)	
n-Hexane		B	29.47 (36.21)	15.41 (26.68)	253.37 (288.16)	
Heptane		B	5.30 (4.88)	3.52 (6.09)	10.94 (3.38)	
4,5-Dimethyl-octane		B	1.52 (1.37)	nd ^4^	2.82 (2.63)	
5-Methyl-nonane		B	1.25 (1.20)	2.66 (2.68)	3.42 (1.54)	
Octane	804	B	3.28 (2.93)	3.93 (1.44)	5.42 (1.19)	
(1-Methylethyl)-cyclopropane	1107	B	1.54 (1.34)	nd	nd	
2-Methyl-azetidine	1252	B	6.85 (2.65)	6.61 (1.28)	7.82 (1.06)	
Nitrosamine						
Nitroso-methane	870	B	14.52 (2.60) ^a^	60.41 (6.05) ^b^	15.51 (0.94) ^a^	139.82 (<0.001)
Aldehydes						
Total aldehydes			190.57 (110.66)	242.27 (96.13)	259.37 (45.61)	
2-Methyl-propanal	807	A	17.63 (18.52)	30.41 (24.24)	17.26 (3.15)	
Propanal	816	B	6.84 (2.30)	7.78 (1.79)	10.05 (1.55)	
2-Methyl-butanal	858	B	32.76 (20.87)	51.62 (23.24)	45.38 (9.60)	
3-Methyl-butanal	860	B	59.57 (36.92)	85.07 (38.07)	75.28 (14.96)	
Pentanal	863	B	6.79 (5.88)	nd	10.47 (1.68)	
Hexanal	1072	A	58.03 (25.12)	57.25 (8.03)	86.77 (14.49)	
Nonanal	1392	A	8.96 (2.64)	7.46 (0.94)	10.18 (0.73)	
2,4-Decadienal	1800	A	nd	2.67 (0.08) ^a^	3.98 (0.30) ^b^	53.15 (0.002)
Ketones						
Total ketones			15.22 (3.40) ^a^	26.62 (2.77) ^ab^	40.07 (8.23) ^b^	16.08 (0.004)
1-(3-Cyclopenten-1-yl)-2-pentanone	811	B	0.49 (0.43)	nd	nd	
Acetone	823	A	nd	nd	18.17 (3.39)	
4-Methyl-3-oxolanone	853	B	2.42 (0.82)	nd	3.71 (0.82)	
2,3-Pentanedione	1050	A	3.62 (1.36) ^a^	7.47 (1.68) ^b^	5.44 (0.65) ^ab^	6.56 (0.031)
Dihydroxyacetophenone	1249	B	nd	nd	1.83 (1.59)	
1-(2-Furanyl)-ethanone	1484	A	4.22 (0.50) ^a^	12.01 (1.42) ^c^	8.56 (0.81) ^b^	46.67 (<0.001)
2,3-Dioxo-4-phenyl butyrolactone	1498	B	2.38 (0.28) ^a^	3.45 (0.06) ^b^	nd	41.69 (0.003)
Maltol	1953	A	1.43 (0.11) ^a^	3.69 (0.33) ^b^	1.37 (0.21) ^a^	96.91 (<0.001)
Terpenes						
Total terpenes			218.60 (76.21) ^a^	172.75 (27.93) ^a^	344.81 (13.69) ^b^	10.55 (0.011)
α-Pinene	1015	A	26.91 (14.35)	21.89 (6.27)	41.06 (1.30)	
α-Thujene	1020	A	19.90 (8.58)	16.06 (4.00)	29.01 (1.98)	
β-Pinene	1091	A	33.12 (14.43) ^ab^	25.75 (5.99) ^a^	51.00 (3.15) ^b^	5.97 (0.037)
β-Sabinene	1106	A	35.26 (12.50) ^ab^	24.13 (5.50) ^a^	50.71 (5.24) ^b^	7.49 (0.023)
α-Terpinene	1130	A	2.34 (2.14)	nd	nd	
3-Carene	1139	A	22.45 (6.77) ^a^	24.27 (3.10) ^a^	40.23 (6.82) ^b^	8.44 (0.018)
α-Sabinene	1157	B	nd	nd	11.69 (0.04)	
Isoterpinolene	1172	B	nd	nd	4.80 (0.16)	
D-Limonene	1192	A	47.56 (11.08) ^a^	38.52 (3.66) ^a^	75.83 (3.96) ^b^	22.44 (0.002)
α-Phellandrene	1199	A	9.17 (2.74) ^ab^	5.81 (0.45) ^a^	12.27 (2.11) ^b^	7.74 (0.022)
β-Terpinene	1243	A	4.78 (2.47) ^ab^	3.63 (0.90) ^a^	8.20 (0.36) ^b^	7.19 (0.026)
Caryophyllene	1600	A	17.12 (2.33) ^ab^	12.68 (0.52) ^a^	20.02 (2.41) ^b^	10.67 (0.011)
Sulphur compounds						
Total sulphur compounds			181.84 (64.75)	119.11 (41.06)	214.07 (35.35)	
Hydrogen sulphide		B	1.82 (0.89)	nd	1.64 (0.34)	
Methanethiol		B	10.71 (5.89)	4.90 (1.66)	6.83 (0.86)	
Dimethyl sulphide	807	A	0.98 (0.62)	1.22 (0.31)	1.26 (0.05)	
1-Propanethiol	830	A	5.23 (3.64)	4.02 (2.01)	7.67 (2.40)	
Methyl-thiirane	847	A	128.63 (51.83)	63.13 (29.25)	147.59 (26.86)	
3-(Methylthio)-1-propene	877	A	2.84 (1.16)	3.70 (0.46)	3.39 (0.63)	
Dimethyl disulphide	1061	A	5.83 (2.86)	10.96 (3.51)	9.52 (1.33)	
Methyl 2-propenyl disulphide	1277	A	10.17 (3.53)	15.22 (1.51)	15.50 (2.02)	
Dimethyl trisulphide	1375	A	2.29 (0.94)	4.46 (1.16)	3.24 (0.12)	
Diallyl disulphide	1467	A	8.52 (3.27)	6.11 (0.74)	10.68 (1.80)	
C6 H10 S2	1472	B	1.19 (0.38)	1.20 (0.35)	1.73 (0.38)	
Methyl 2-propenyl trisulphide	1585	A	3.06 (1.08)	3.82 (0.56)	4.27 (0.51)	
Allyl trisulphide	1784	A	0.57 (0.24)	0.36 (0.09)	0.74 (0.12)	
Furan						
Total furan			23.99 (5.72) ^a^	55.62 (4.93) ^b^	43.99 (5.51) ^b^	26.38 (0.001)
3-Methyl-furan	842	A	5.69 (2.98)	6.19 (1.13)	3.47 (0.89)	
2,3-Dihydro-4-methyl-furan	1082	B	nd	4.03 (0.23)	4.99 (0.92)	
2-Pentyl-furan	1227	A	6.55 (2.00)	7.39 (1.27)	7.26 (1.05)	
Furfural	1448	A	11.75 (0.89) ^a^	28.14 (3.18)	22.14 (2.51) ^b^	35.96 (<0.001)
2-Furanmethanol	1659	A	nd	9.87 (0.61) ^b^	6.13 (0.44) ^a^	72.79 (0.001)
Pyrazines						
Total pyrazines			33.52 (3.74) ^a^	124.76 (14.12) ^c^	80.11 (6.01) ^b^	75.04 (<0.001)
Pyrazine	1202	A	nd	5.92 (0.56) ^b^	4.78 (0.26) ^a^	10.16 (0.033)
Methyl-pyrazine	1264	A	28.70 (2.62) ^a^	70.69 (8.83) ^c^	46.31 (3.85) ^b^	40.15 (<0.001)
2,5-Dimethyl-pyrazine	1322	A	nd	8.06 (0.88)	nd	
2,6-Dimethyl-pyrazine	1329	A	4.82 (1.13) ^a^	12.41 (0.78) ^c^	7.26 (0.56) ^b^	61.27 (<0.001)
Ethyl-pyrazine	1334	A	nd	12.39 (1.13) ^b^	8.01 (0.99) ^a^	25.53 (0.007)
2,3-Dimethyl-pyrazine	1347	A	nd	5.30 (0.56) ^b^	2.85 (0.33) ^a^	42.46 (0.003)
2-Ethyl-6-methyl-pyrazine	1384	A	nd	4.37 (0.61) ^b^	2.63 (0.55) ^a^	13.44 (0.021)
2-Ethyl-3-methyl-pyrazine	1401	A	nd	5.61 (0.92) ^b^	3.26 (0.40) ^a^	16.70 (0.015)
Pyrimidine						
4,6-Dimethyl-pyrimidine	1322	B	nd	nd	5.01 (0.36)	
*Benzene*						
1-Ethyl-2,3-dimethyl-benzene	1268	B	21.30 (6.83)	18.26 (1.40)	28.36 (1.40)	
Esters						
Total esters			4.78 (1.17) ^a^	26.31 (5.79) ^b^	22.02 (1.65) ^b^	31.11 (0.001)
Ethyl isobutyrate	882	B	2.08 (0.05)	2.54 (1.17)	1.58 (0.36)	
2-Propynyl propionate	1104	B	nd	17.68 (4.05)	15.21 (1.04)	
Methyl acetate	1294	B	3.39 (0.04) ^a^	6.08 (0.85) ^b^	3.88 (0.17) ^a^	24.91 (0.001)
Octyl formate	1549	A	nd	nd	1.35 (0.13)	
Acids						
Total acids			13.92 (4.68) ^a^	27.56 (2.14) ^b^	21.94 (2.73) ^b^	12.47 (0.007)
Propiolic acid		B	nd	nd	10.30 (1.78)	
2-Acetoxysuccinic acid	886	B	nd	12.99 (3.14)	nd	
Acetic acid	1439	A	5.98 (1.20) ^a^	9.03 (1.03) ^b^	5.06 (0.71) ^a^	12.86 (0.007)
Hexanoic acid	1827	A	7.94 (4.16)	5.55 (1.97)	6.57 (2.36)	

^1^ LRI: Linear Retention Index calculated on a DB-Wax stationary phase with a series of alkanes between C8 and C26. ^2^ ID, method of identification: A = mass spectrum and LRI references, B = tentative identification by mass spectrum only. ^3^ Results from ANOVA (only those with significant differences are shown). For each row, different superscript denotes a significant difference (*p* < 0.05). ^4^ nd = not detected.

**Table 8:** Intensity of sensorial attributes (from RATA questions) and overall liking of fried nuggets: mean (SD).

Attribute	NO	NP	NV	F-Value (*p*-Value)
Toasted appearance	1.77 (0.81) ^a^	2.23 (0.71) ^b^	1.96 (0.71) ^ab^	6.21 (0.003)
Visual crunchy	2.61 (0.53)	2.68 (0.51)	2.57 (0.50)	0.78 (0.462)
External homogeneity	2.23 (0.76)	2.05 (0.77)	2.00 (0.74)	1.96 (0.147)
Internal homogeneity	2.16 (0.68)	2.09 (0.72)	2.21 (0.68)	0.60 (0.540)
Spice odour	1.21 (0.97)	0.98 (0.88)	1.20 (1.02)	1.32 (0.272)
Off odour	0.11 (0.31)	0.14 (0.40)	0.14 (0.44)	0.22 (0.780)
Fried odour	1.77 (0.79) ^ab^	2.04 (0.99) ^b^	1.64 (0.82) ^a^	3.99 (0.022)
Crunchy exterior	2.54 (0.63)	2.70 (0.54)	2.61 (0.59)	1.23 (0.297)
Juiciness	2.11 (0.76)	1.93 (0.76)	2.18 (0.79)	2.01 (0.143)
Chewability	2.55 (0.63)	2.38 (0.62)	2.50 (0.50)	1.82 (0.169)
Oily texture	1.14 (0.92)	1.16 (0.89)	1.18 (0.79)	0.04 (0.956)
Chicken flavour	1.62 (0.82)	1.61 (0.80)	1.73 (0.82)	0.63 (0.512)
Oily flavour	1.09 (0.77)	1.11 (0.87)	1.00 (0.83)	0.50 (0.610)
Spice flavour	1.30 (0.99)	1.23 (0.83)	1.52 (0.91)	1.89 (0.157)
Burnt flavour	0.62 (0.89) ^ab^	0.96 (1.03) ^b^	0.52 (0.76) ^a^	4.70 (0.013)
Off flavour	0.30 (0.60)	0.25 (0.51)	0.36 (0.77)	0.50 (0.605)
Acid aftertaste	0.16 (0.46)	0.23 (0.54)	0.32 (0.61)	1.88 (0.158)
Overall Liking	6.71 (1.42)	6.41 (1.53)	6.82 (1.62)	1.23 (0.298)

For each row, different superscript denotes a significant difference (*p* < 0.05).

## Data Availability

The data that support the findings of this study are openly available in https://doi.org/10.5281/zenodo.17104978.
